# Concerning Criminals

**Published:** 1889-03

**Authors:** S. H. Preston


### CONCERNING CRIMINALS.

BY S. H. PRESTON.

Though the old Calvinistic notion that certain babies are born as unadulterated compounds of Adamic depravity has about been banished from the realms of religious absurdity, it is still an indisputable fact that there are children launched into life with an inherited inclination for iniquity. Not that they take to wrong doing for the delight of doing wrong, but the transmitted taint of a thousand generations seems to give them a headlong trend down the career of crime.

There are probably beings born with as special tendency to sin as others are to scrofula. There is a virus of vice in their moral make-up for which they are no more accountable than for bad blood in their bodies. They are what may be truly termed foetal felons—children conceived in criminal impulse, or maybe imbibing the malice of murder in their mother's milk. Poor guiltless victims of parental ignorance or gestational conditions ! Their only crime is in being born, in coming into the world as criminals through the cruelty of circumstances ; yet it is a crime the world will not condone and fearful are the punishments it frequently inflicts.

Oh, yes, of course, society should protect itself, but not as the hunter with hounds in pursuit of the rabbit or the hare. The world is wide enough and mankind mighty enough to quarantine every earthly evil—to put every criminal in probationary isolation, to exhaust every resource of experimental reformation, before having recourse to cruel and retaliatory punishment. It has not been conclusively shown that even the worst felon that ever lived was hopelessly beyond the pale of human reclamation.

All that society has a right to ask is safety, not judicial vengeance. The deterrent influence of the death penalty has never been apparent. Indeed, murder was more common when men were hung for stealing a shilling and heads were set on temple bar. Crime was no less prevalent when people were burnt alive for theologic beliefs. Make *Autas-da-fe* as common in Madison Square as once they were in Madrid, and the callous crowd would soon care no more for them than for a street-car strike. A spectacle of brutality but serves to brutalize the beholders. The public sentiment of the times that prohibits its infliction upon horses still permits human beings to be hung up by the neck—still suffers women to be strangled upon the scaffold.

Time was when folks afflicted with disease were put out of the way, not as Bergh's men kindly kill a disabled animal to mercifully end its distress, but because deemed unfit to live. Now the crowning glory of our century is its magnificent hospitals Time will be when our prisons, the disgrace of this grand age, will be changed to humanitarian institutions for the control and cure of crime, instead of pest places for its punishment and propagation.

Statesmen and reformers are beginning to consider that our system of society is really accountable for more crime than it prevents Judges and juries are frequently called upon to condemn unfortunate creatures of circumstances less guilty of offence against their fellows than those before whom they are arraigned. Did all wrong-doers escape detection, as doubtless the majority do, there would be no criminals known ; while if the consciences of men were encased in glass all would be criminals alike. There would be no honest men to bring the guilty to justice. Let all who read reflect upon this.

He who robs you wrongs himself worse than you. You may recover your property or purchase more, but he has corrupted his conscience, ruined his reputation, assassinated his manhood. He has inflicted upon himself a misfortune, while you have but sustained a pecuniary and perchance, a trifling loss.

The treatment of crime is truly a solemn subject. Its commission is most frequently the effect of ignorance, folly, unpremeditated parley with temptation, or unfortunate relations of life. Our criminal courts, instead of being besieged by idle sight-seers, often to find material for merriment, should be thronged by the philanthropic and the Christ-like to render what remedial help they can to the human wrecks drifting upon the rocks of crime.

"What might be done if men were wise!
What glorious deeds, my suffering brother,
Would they unite
In love and right,
And cease their scorn of one another.

The meanest wretch that ever trod,
The deepest sunk in guilt and sorrow,
Might stand erect
In self-respect,
And share the teeming world to-morrow."

The old vindictive method of dealing with crime has had a thorough trial and crime is still increasing at an appalling rate. Now, let society seek a system of prevention and reformation rather than one of punishment and condemnation—employ its magnificent moral machinery in providing for the propagation of better beings, stop the supply of criminals from the cradle and permit the sunshine of a beneficent civilization to pervade our prisons and penitentiaries.

---

Hay Fever.—Dr. Morell Mackenzie, in his monograph on this complaint and its treatment, says that, among races, the English and American; among classes, the upper and cultivated; and of the sexes, the males are especially susceptible to hay fever. In the north of Europe it is almost unknown. It is rare in France, Germany, Italy and Spain; whereas in England it is frequent, and in America prevalent. Again, 99 per cent. of its martyrs are of the upper class, while agricultural laborers, who are most exposed to the cause of the complaint, are less subject to its attacks. Lastly, the male sex is more liable to it than the female, in the ratio of three to one. He gives its cause—"the entrance into the eyes and air-channels of those predisposed to the ailment, of minute particles of vegetable matter from grasses and plants in flower"—and its cure, chiefly cocaine in one form or another, or residence in certain mountain or seashore localities which are free from the disease.

